# First report of *Haemaphysalis bispinosa*, molecular-geographic relationships of *Ixodes granulatus* and a new *Dermacentor* species from Vietnam

**DOI:** 10.1186/s13071-024-06641-7

**Published:** 2025-01-23

**Authors:** Sándor Hornok, Jenő Kontschán, Gergő Keve, Nóra Takács, Dat Van Nguyen, Khanh Ngoc Phuong Ho, Tamás Görföl, Yuanzhi Wang, Róbert Farkas, Thanh Thi Ha Dao

**Affiliations:** 1 Department of Parasitology and Zoology, University of Veterinary Medicine, Budapest, Hungary; 2 HUN-REN-UVMB Climate Change: New Blood-Sucking Parasites and Vector-Borne Pathogens Research Group, Budapest, Hungary; 3 Plant Protection Institute, HUN-REN Centre for Agricultural Research, Martonvásár, Hungary; 4 Department of Plant Sciences, Albert Kázmér Faculty of Mosonmagyaróvár, Széchenyi István University, Mosonmagyaróvár, Hungary; 5 National Institute of Malariology, Parasitology and Entomology, Hanoi, Vietnam; 6 Endangered Primate Rescue Center, Cuc Phuong National Park, Ninh Binh, Vietnam; 7 National Laboratory of Virology, Szentágothai Research Centre, University of Pécs, Pécs, Hungary; 8 Key Laboratory for Prevention and Control of Emerging Infectious Diseases and Public Health Security, the XPCC, School of Medicine, Shihezi University, Shihezi, Xinjiang, Uygur Autonomous Region China; 9 Department of Parasitology, National Institute of Veterinary Research, Hanoi, Vietnam

**Keywords:** Host association, Geographical pattern, *Cox*1, 16S rRNA gene

## Abstract

**Background:**

Vietnam and its region are regarded as an ixodid tick biodiversity hotspot for at least two genera: *Haemaphysalis* and *Dermacentor*. To contribute to our knowledge on the tick fauna of this country, ticks from these two genera as well as an *Ixodes* species were analyzed morphologically and their molecular-phylogenetic relationships were examined in taxonomic and geographical contexts.

**Methods:**

For this study, seven *Haemaphysalis* sp. ticks were removed from dogs and collected from the vegetation. These showed morphological differences from congeneric species known to occur in Vietnam. In addition, three *Ixodes* sp. ticks were collected from pygmy slow lorises (*Xanthonycticebus pygmaeus*), and a *Dermacentor* female had been previously collected from the vegetation. After DNA extraction, these were molecularly or phylogenetically analyzed based on the cytochrome *c* oxidase subunit I (*cox*1) and 16S rRNA genes.

**Results:**

The three species were morphologically identified as (i) *Ixodes granulatus*, which had nearly or exactly 100% sequence identities to conspecific ticks reported from large (approximately 2000 km) geographical distances but was more different (having lower, only 94.2% *cox*1 and 96.7% 16S rRNA sequence identity) from samples collected within 1000 km of Vietnam in Southern China and Malaysia, respectively; (ii) *Haemaphysalis bispinosa*, which showed 100% sequence identity to samples reported within both narrow and broad geographical ranges; and (iii) a new species, *Dermacentor pseudotamokensis* Hornok sp. nov., described here morphologically and shown to be phylogenetically a sister species to *Dermacentor tamokensis*.

**Conclusions:**

*Haemaphysalis bispinosa* shows genetic homogeneity in the whole of South and Southeast Asia, probably owing to its frequent association with domestic ruminants and dogs (i.e. frequently transported hosts). However, *I. granulatus*, the Asian rodent tick, has a mixed geographical pattern of haplotypes, probably because it may associate with either synanthropic or wild-living rodents as primary hosts. This tick species is recorded here, for the first time to our knowledge, as parasitizing lorises in Vietnam and its region. Based on phylogenetic analyses, *D. pseudotamokensis* Hornok sp. nov., recognized and described here for the first time, was almost certainly misidentified previously as *Dermacentor steini*, drawing attention to the need to barcode all *Dermacentor* spp. in Southern Asia.

**Graphical Abstract:**

**Figure:**
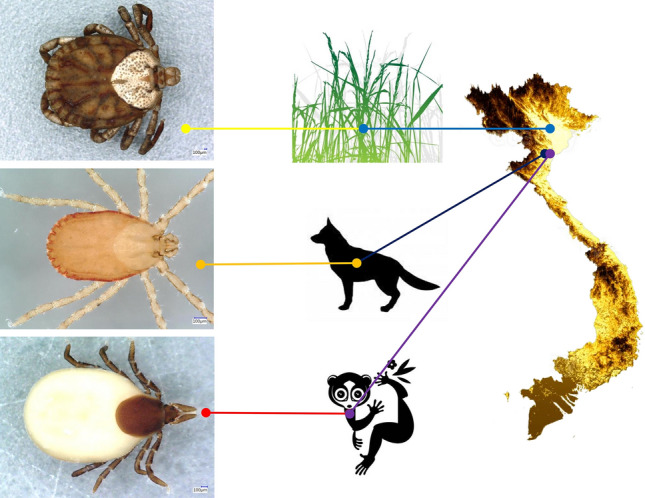

**Supplementary Information:**

The online version contains supplementary material available at 10.1186/s13071-024-06641-7.

## Introduction

The Oriental Zoogeographic Region extends from Pakistan and the Indian subcontinent in the west to southern Japan (Ryukyu Islands) in the east and includes southern China, the Indochinese Peninsula and some parts of Indonesia [1]. This vast geographical area has both temperate and tropical climate zones, ensuring higher-than-average biodiversity, as also exemplified by hard tick species (Acari: Ixodidae) indigenous to this region [1]. In particular, discounting the Afrotropical Region, the Oriental Region provides suitable habitats for the highest number (*n* = 185) of tick species, and among hard tick genera, both *Haemaphysalis* and *Dermacentor* species are the most numerous in this zoogeographical region, with 98 and 16 species, respectively. In the Oriental Region, Vietnam has the third highest number of ixodid tick species, following China and India in decreasing order [1].

Until recently, 62 tick species were regarded as indigenous to Vietnam [2]. This included only six species from the genus *Ixodes*, which contains the most hard tick species worldwide [3]. However, recently two further species, *Ixodes lanigeri* and *Ixodes abramovi*, were discovered as new to science and described from Vietnam [4, 5], thus increasing the number of *Ixodes* species occurring in this country from six to eight. However, 30 *Haemaphysalis* species were long known to occur in Vietnam, and one was recently added to the fauna from this second-most species-rich genus, for which Southeast Asia is regarded as a biodiversity-evolutionary hotspot [2, 6]. These data, as well as descriptions of seven new *Dermacentor* species from the past decade based on samples collected in Vietnam or its region [7–14], highlight the importance of this country for tick species diversity.

In this study, one member of each of these three important hard tick genera was studied for morphological details and to obtain their hitherto missing molecular-phylogenetic data. Relevant to these three tick species, although they occur in other countries in Southeast Asia, no high-quality digital microscopical pictures have been available so far, to our knowledge, to illustrate their morphology and aid in their identification, which is now provided by this study. In addition, a new *Dermacentor* species is described here for the first time, clarifying its likely previous misidentification both inside and outside Vietnam.

## Methods

### Sample sources and morphological identification

The following specimens were selected and used in this study from ticks collected in Vietnam:

(i) *Ixodes granulatus* females (*n* = 3) were collected from rescued pygmy slow lorises (*Xanthonycticebus pygmaeus*) sampled on March 22, 2023, in Thanh Liem Commune, Phu Ly District, Ha Nam Province (*n* = 1), and on April 14, 2023, in Thuong Xuan District, Thanh Hoa Province (*n* = 2), Vietnam.
(ii) *Haemaphysalis bispinosa* males (*n* = 2) and females (*n* = 4) were collected from female dogs (*Canis lupus var. familiaris*) during regular veterinary care in Cuc Phuong Commune, Nho Quan District, Ninh Binh Province, Vietnam. In addition, a single nymph of this species was collected at the same location from the vegetation by the dragging-flagging method on March 28, 2024.
(iii) A *Dermacentor* sp. female was collected from the vegetation on October 2, 2016, at the Me Linh Research Station, Phuc Yen, Vinh Phuc Province, Vietnam.

These ticks were stored in 96% ethanol, and their species were identified (i) based on Yamaguti et al. [15], (ii) adults according to Tanskull et al. [16] and the nymph based on Kwak and Ng [17] and (iii) compared with Apanaskevich and Apanaskevich [11], respectively. Pictures were taken with a Keyence VHX-5000 digital microscope (Osaka, Japan).

### Molecular and phylogenetic analyses

These were performed as described [2]. In brief, DNA was extracted with the QIAamp DNA Mini Kit (QIAGEN, Hilden, Germany), including an overnight digestion in tissue lysis buffer and Proteinase K at 56 °C. PCR amplification of an approximately 710-bp-long part of the cytochrome *c* oxidase subunit I (*cox*1) gene was performed with the primers LCO1490 (forward: 5’-GGT CAA CAA ATC ATA AAG ATA TTG G-3’) and HCO2198 (reverse: 5’-TAA ACT TCA GGG TGA CCA AAA AAT CA-3’). The reaction mixture contained 1 U (0.2 µl) HotStarTaq Plus DNA polymerase, 2.5 µl 10 × CoralLoad Reaction buffer (with 15 mM MgCl_2_), 0.5 µl PCR nucleotide mix (0.2 mM each), 0.5 µl (1 µM final concentration) of each primer, 15.8 µl ddH_2_O and 5 µl template DNA in a volume of 25 µl. The PCR included an initial denaturation step at 95 °C for 5 min, followed by 40 cycles of denaturation at 94 °C for 40 s, annealing at 48 °C for 1 min and extension at 72 °C for 1 min. Final extension was done at 72 °C for 10 min. Another PCR was used to amplify an approximately 460-bp fragment of the 16S rDNA gene of Ixodidae, with the primers 16S + 1 (5’-CTG CTC AAT GAT TTT TTA AAT TGC TGT GG-3’) and 16S-1 (5’-CCG GTC TGA ACT CAG ATC AAG T-3’). Reaction component, cycling conditions were the same as above, except for annealing at 51 °C. Purification and sequencing of the PCR products were done by Eurofins Biomi Ltd. (Gödöllő, Hungary). Quality control and trimming of sequences were performed with the BioEdit program. Obtained sequences were compared to GenBank data by the nucleotide BLASTN program (https://blast.ncbi.nlm.nih.gov). New sequences were submitted to GenBank under the following accession numbers (cytochrome *c* oxidase subunit I [*cox*1] gene: PQ439197-PQ439201, 16S rRNA gene: PQ453065-PQ453069). During phylogenetic analyses, sequence datasets were resampled 1000 times to generate bootstrap values. Phylogenetic analyses were performed with the neighbor-joining method, p-distance model in MEGA11 [18].

## Results

### Morphological identification of tick species

**Fig. 1:**
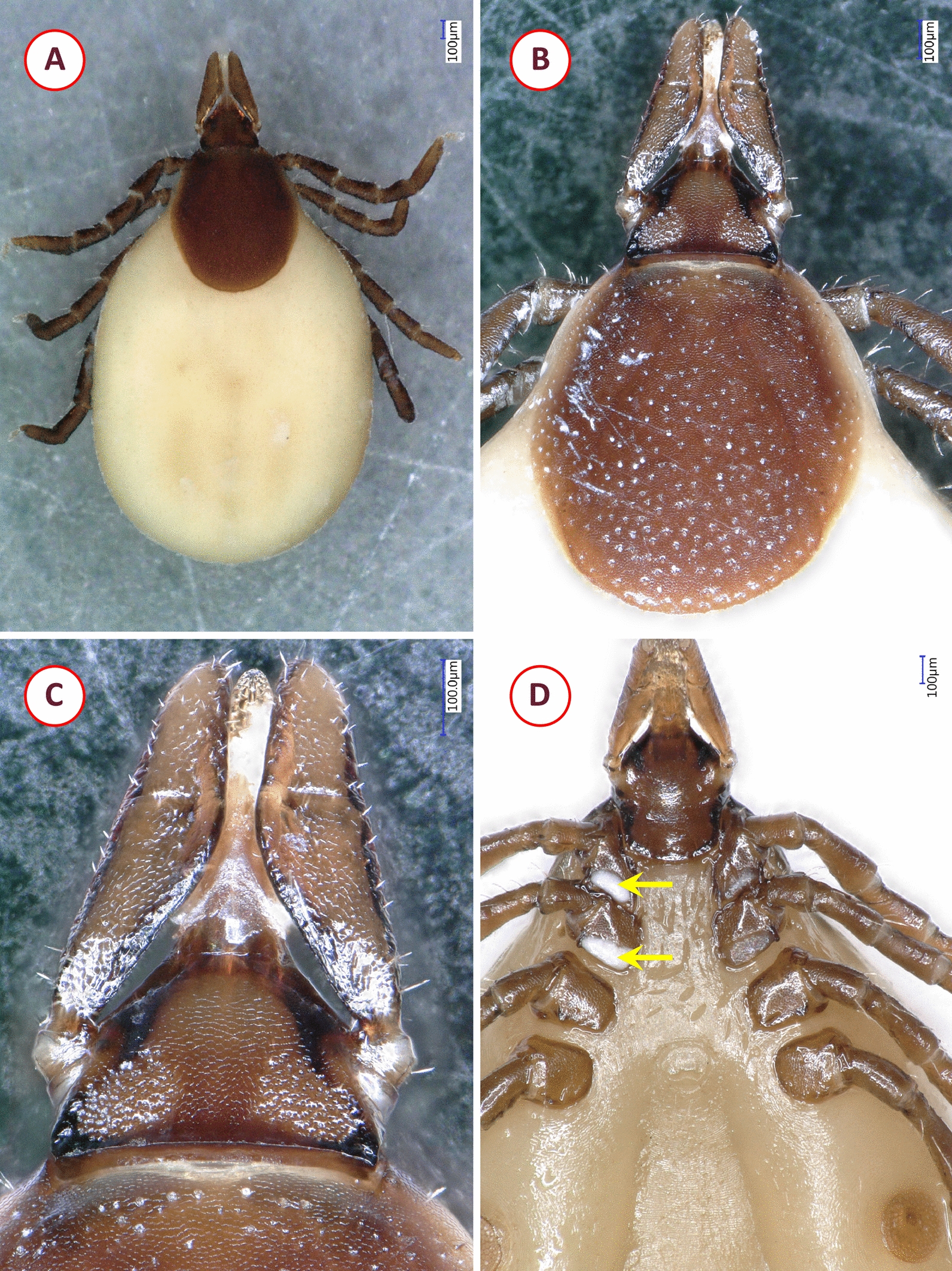
Morphological characters of *Ixodes granulatus* female collected in Vietnam: **A** dorsal view (habitus); **B** scutum and dorsal view of basis capituli, palps; **C** enlarged dorsal view of basis capituli, palps; **D** ventral view showing spiracular plate, coxae (arrows indicate syncoxae), basis capituli and palps

*Ixodes granulatus* females were identified based on the following characters: ovoid scutum with dense, deep punctuations; posteriorly almost straight cervical grooves (fading towards a concavity in the posterolateral scutal margin) and lateral carinae; shape of basis close to an equal-sided triangle, without cornua but sharp, acute-angled caudolateral corners; interval separating porose areas equal to their width; straight lateral and obtuse-angled medial edge of palps; ventrally on the basis short ridge in place of auriculae; presence of I-II syncoxae; genital aperture between coxae IV; spiracular plates rounded (Fig. 1).

**Fig. 2:**
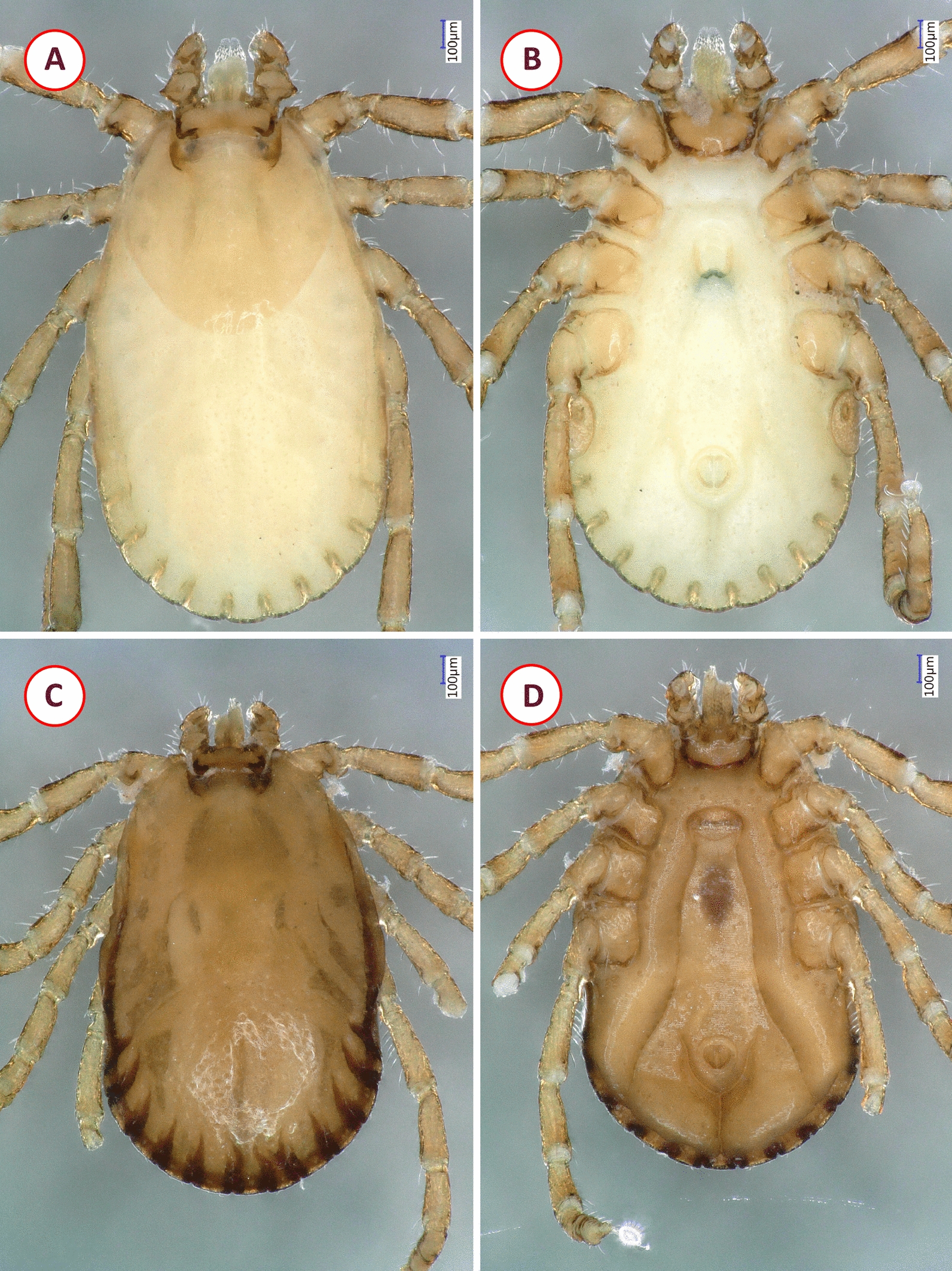
Morphological characters of *Haemaphysalis bispinosa* adults collected in Vietnam: **A** dorsal view of female; **B** ventral view of female; **C** dorsal view of male; **D** ventral view of male

**Fig. 3:**
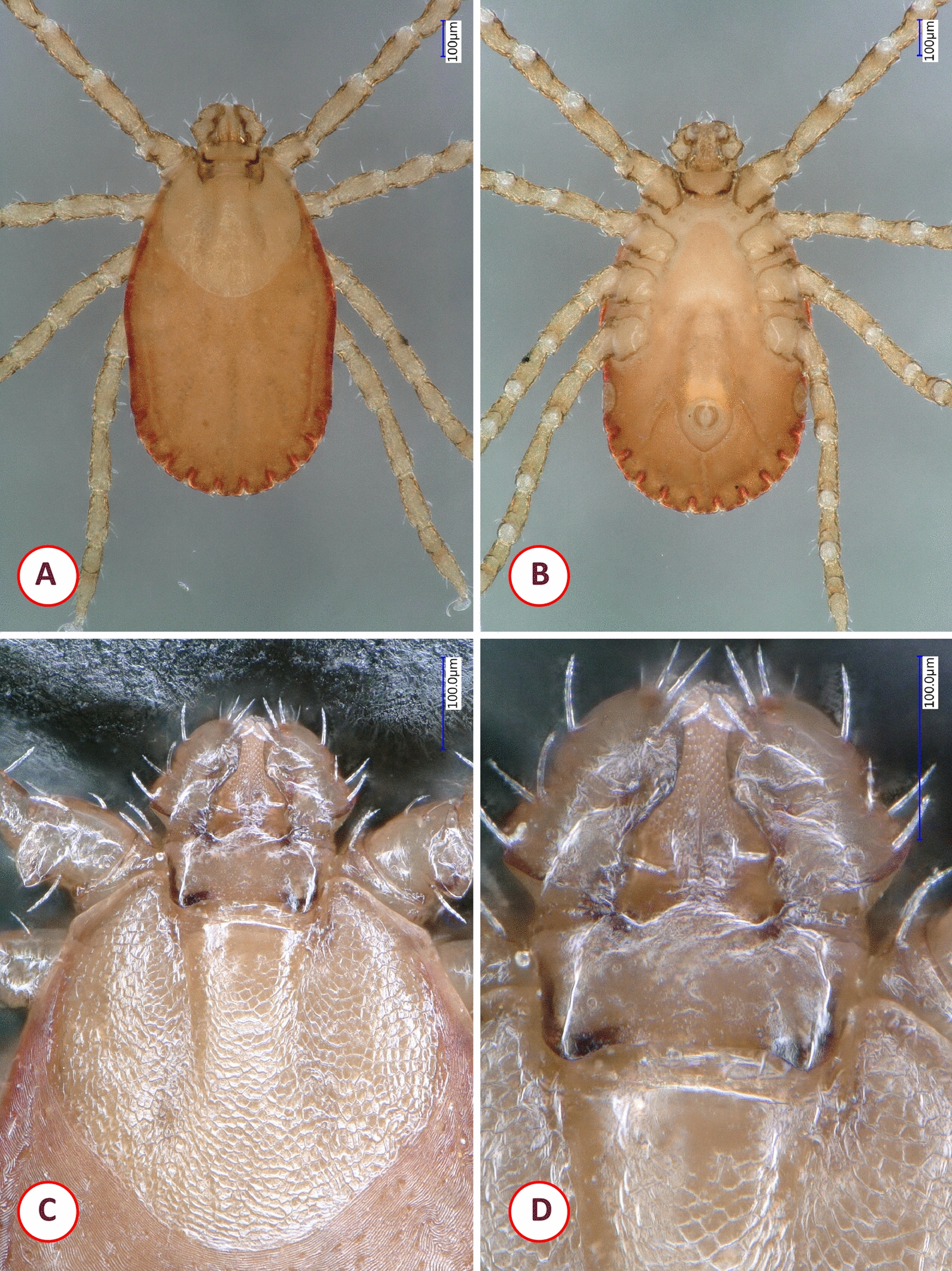
Morphological characters of *Haemaphysalis bispinosa* nymph collected in Vietnam: **A** dorsal view; **B** ventral view; **C** scutum and dorsal view of basis capituli, palps; **D** enlarged dorsal view of basis capituli, palps

*Haemaphysalis bispinosa* males had distinct lateral grooves extending from anterior margin of festoon I to level of coxa II, broadly triangular ventral spur on palpal segment III; long spur on coxae I and short spur on coxae II–IV. Females had short, slightly curved cervical grooves, palpal and coxal spurs as in male; in both sexes backward pointing cornua (Fig. 2). The nymph showed medial edge of palpal segment III slightly extending internally beyond medial edge of palpal segment II; lateral edge of palpal segment II narrow; cornua and spurs as in adults (Fig. 3).

The morphology of the *Dermacentor* sp. collected in Vietnam was most similar to that of *Dermacentor tamokensis*, but it was different from the latter based on the scutum, basis capituli, palps, spiracular plates and genital pore (see below: Description of the new *Dermacentor* species).

### Molecular-phylogenetic analysis of tick species

**Fig. 4:**
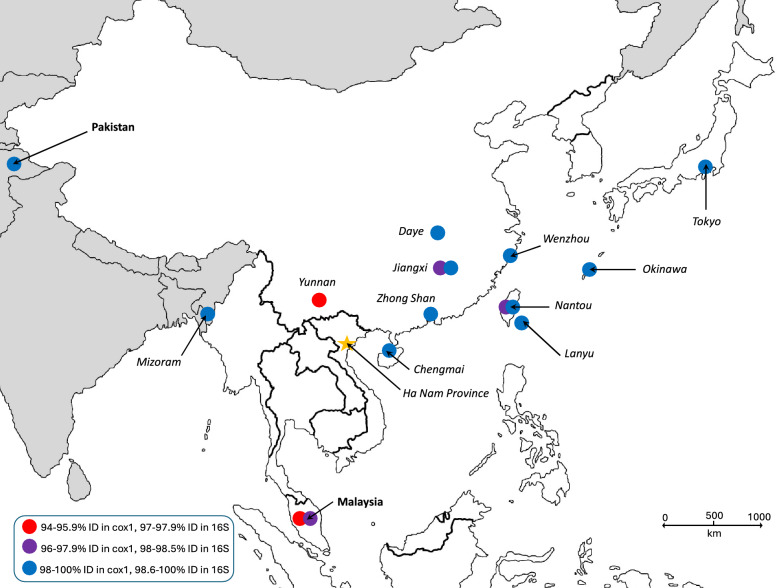
Map of South-Southeast and East Asia illustrating the geographical distribution of *Ixodes granulatus* mitochondrial haplotypes according to sequence heterogeneity

The three *I. granulatus* females collected from lorises in Vietnam had 2–4 bp (up to 0.6%) *cox*1 sequence differences (638–640/642 bp identity) compared with each other (PQ439199–PQ439201). These were also 98.9–99.8% (635-/642 to 452/453 bp) identical to the majority of sequences reported as those of *I. granulatus* from other countries of South and Southeast Asia, except one from Yunnan, China (NC_061226, sharing only 605/642 bp, i.e. 94.2% identity with VQ13) and another from Jiangxi, China (MG721051, with 510/525 bp = 97.1% identity). In the 16S rRNA gene, only two of the three *I. granulatus* females from Vietnam had 1-bp difference (PQ453067-PQ453069). Compared with samples from other countries, most had nearly 100% 16S rRNA gene sequence identity to the samples from Vietnam, except samples from Malaysia (MT914179: 414/428 bp, i.e. 96.7% identity) and Taiwan (DQ002995: 417/430, i.e. 97% identity) (Fig. 4, Supplementary Table 1).

All seven *Haemaphysalis bispinosa* specimens collected in northern Vietnam (including those from dogs and from the vegetation) had identical *cox*1 and 16S rRNA haplotypes. These (PQ439198, PQ453066, respectively) showed 100% sequence identity to samples reported within both narrower or broader geographical ranges, i.e. from Java (ON778582), Thailand (OR335055), Bangladesh (MK269314), India (ON416654) and Pakistan (PP325825).

**Fig. 5:**
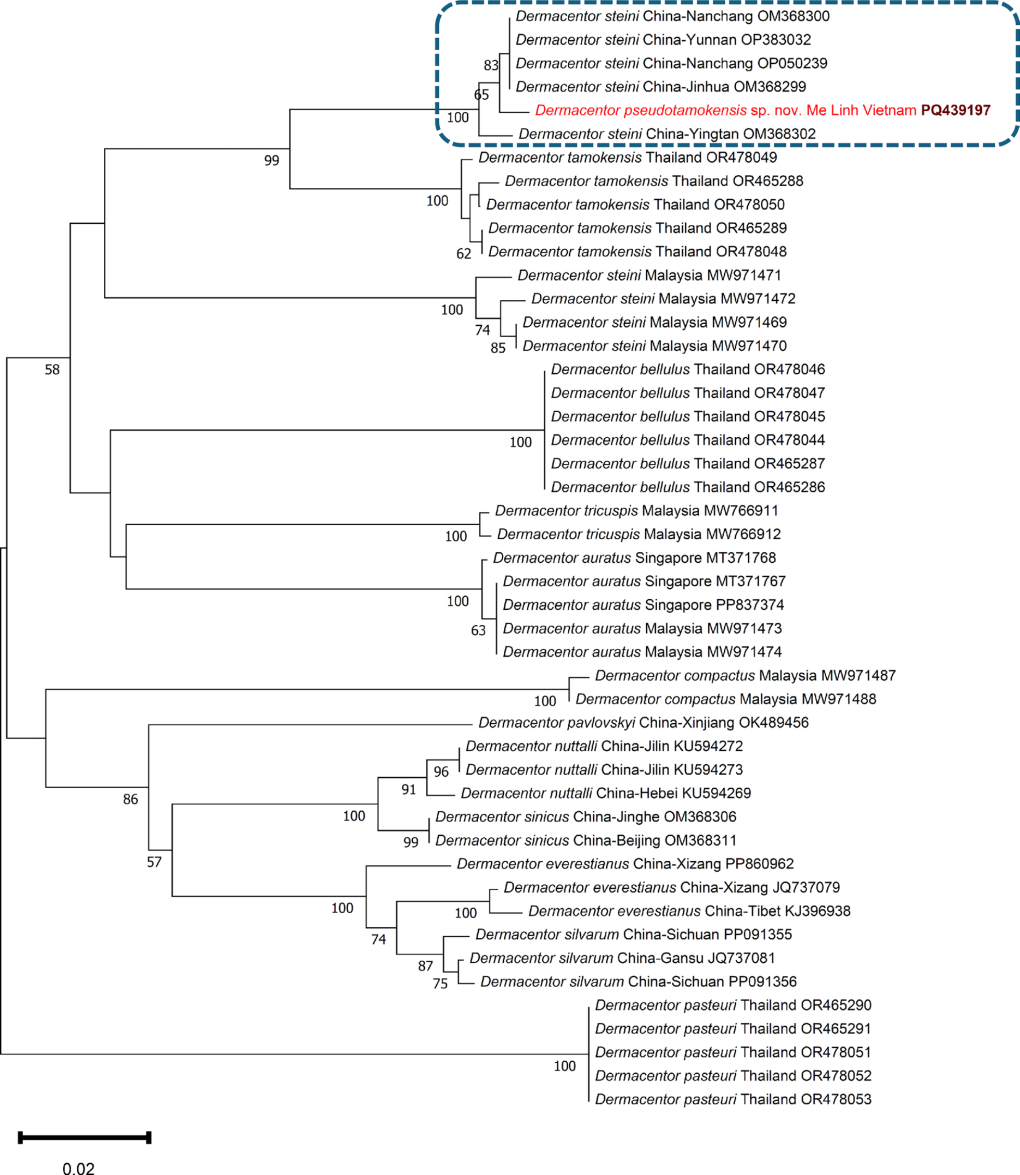
Phylogenetic tree of Southeast Asian *Dermacentor* species based on the *cox*1 gene. In each row of individual sequences, the country of origin and the GenBank accession number are shown after the species name. The sequence from this study is indicated with red fonts and bold, maroon accession numbers. The clade of *Dermacentor pseudotamokensis* sp. nov., which probably includes conspecific ticks, is encircled with blue, dashed line. The evolutionary history was inferred by using the neighbor-joining method and p-distance model. The tree is drawn to scale, with branch lengths measured in the number of substitutions per site. The analysis involved 47 nucleotide sequences, and there were a total of 322 positions in the final dataset. Evolutionary analyses were conducted in MEGA11

**Fig. 6:**
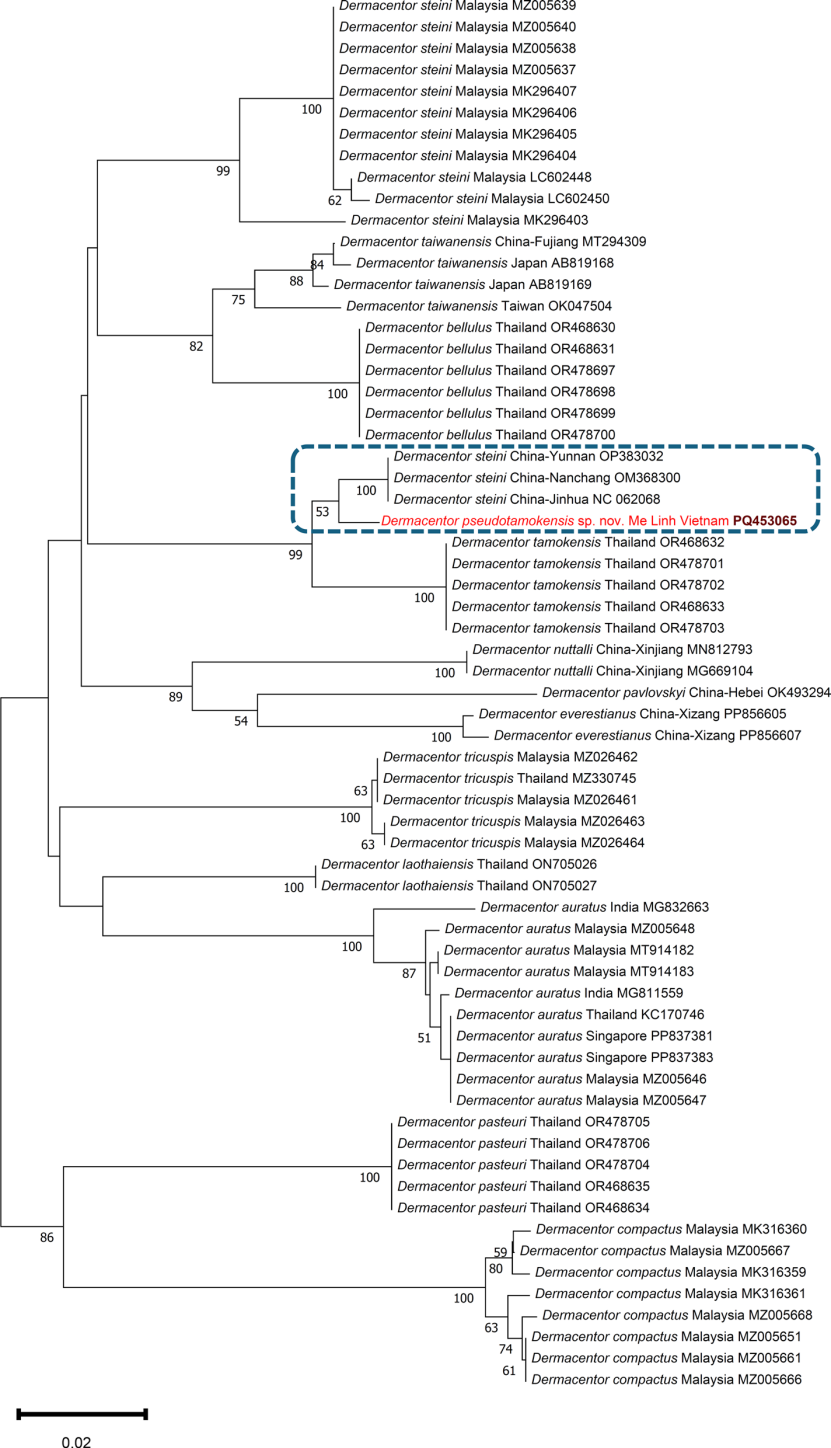
Phylogenetic tree of Southeast Asian *Dermacentor* species based on the 16S rRNA gene. In each row of individual sequences, the country of origin and the GenBank accession number are shown after the species name. The sequence from this study is indicated with red fonts and bold, maroon accession numbers. The clade of *Dermacentor pseudotamokensis* sp. nov., which probably includes conspecific ticks, is encircled with blue, dashed line. The evolutionary history was inferred by using the neighbor-joining method and p-distance model. The tree is drawn to scale, with branch lengths measured in the number of substitutions per site. The analysis involved 65 nucleotide sequences, and there were a total of 372 positions in the final dataset. Evolutionary analyses were conducted in MEGA11

The *cox*1 and 16S rRNA gene sequences (PQ439197, PQ453065) of the *Dermacentor* sp. collected from the vegetation at the Me Linh Research Station (Phuc Yen) showed 98.9–99.5% (635–639/642 bp) and 98.1–98.3% (414–415/422 bp) identity, respectively, to sequences reported under the name *Dermacentor steini* from China (Jinhua: OM368299, Nanchang: OM368300 and OP050239, Yunnan: OP383032, Yingtan: OM368302). At the same time, this *Dermacentor* sp. from northern Vietnam had only 93.5–94% (361–363/386 bp) *cox*1 and 95.8–96% (340/354 to 340/355 bp) 16S rRNA sequence identity to the morphologically most similar species, *D. tamokensis* (OR465288, OR478049 and OR468632, OR468633, respectively). Phylogenetic analyses based on the *cox*1 or 16S rRNA gene showed that the *Dermacentor* sp. from Me Linh Research Station (Phuc Yen) clustered as a sister species to *D. tamokensis*, forming one group with the above specimens reported under the name *D. steini* from East and South China (Figs. 5, 6). These clustered distantly from bona fide *D. steini* published from Malaysia (e.g. MW971471, MZ005638). These data support that it is a new species.

### Description of the new *Dermacentor* species

**Family Ixodidae Koch, 1844**

**Genus** ***Dermacentor *Koch, 1844**

***Dermacentor*** ***pseudotamokensis*** **Hornok sp. nov.**

*Type host*: unknown.

*Other hosts*: Ticks with closely related *cox1* and 16S rRNA haplotypes were found on goats (*Capra aegagrus hircus*) and Sikkim rats (*Rattus andamanensis*) in China (OM368300 and OP383032, respectively).

*Type locality*: Me Linh Research Station (coordinates 21.38N, 105.71E), Phuc Yen, Vinh Phuc Province, Vietnam.

*Other localities*: Ticks with closely related *cox1* and 16S rRNA haplotypes were recorded in South and East China: Jinhua (OM368299), Nanchang (OM368300, OP050239), Yunnan (OP383032) and Yingtan (OM368302).

*Type specimens*: Holotype: female from vegetation, collected by Dr. Ottó Merkl on October 2, 2016, at the Me Linh Research Station (coordinates 21.38N, 105.71E), Phuc Yen, Vinh Phuc Province, Vietnam; deposited at the Department of Parasitology and Zoology, University of Veterinary Medicine, Budapest, Hungary (accession no. UNIVET-PAR-HS213).

*Representative DNA sequences*: Mitochondrial *cox1* and 16S rRNA gene sequences of the holotype are deposited in GenBank under PQ439197 and PQ453065, respectively. Complete mitochondrial genome sequences of ticks with closely related *cox1* and 16S rRNA haplotypes are available in GenBank (accession nos. OM368300, OP383032).

*ZooBank registration*: To comply with the regulations set out in article 8.5 of the amended 2012 version of the *International Code of Zoological Nomenclature* (ICZN) [19], details of the new species have been submitted to ZooBank. The Life Science Identifier (LSID) of the article is urn:lsid:zoobank.org:pub:8E0BF8B8-9DD7-408A-9743-AA64A235A950. The LSID for the new name *Dermacentor pseudotamokensis* is urn:lsid:zoobank.org:act:C7E92DA8-11D6-4EDD-83C0-41F16E4EDF52.

*Etymology*: The name of the new species refers to the most similar species, *D. tamokensis*, at the same time implying its differences.

### Description

*General*: Large size, brownish ornate metastriate tick. Basis capituli dorsally rectangular, with short cornua. Scutum, legs, palps with whitish enamel covering. Scutum heart-shaped, broadest anteriorly, with short, S-shape cervical grooves and scattered, deep, small to large punctuations.

**Fig. 7:**
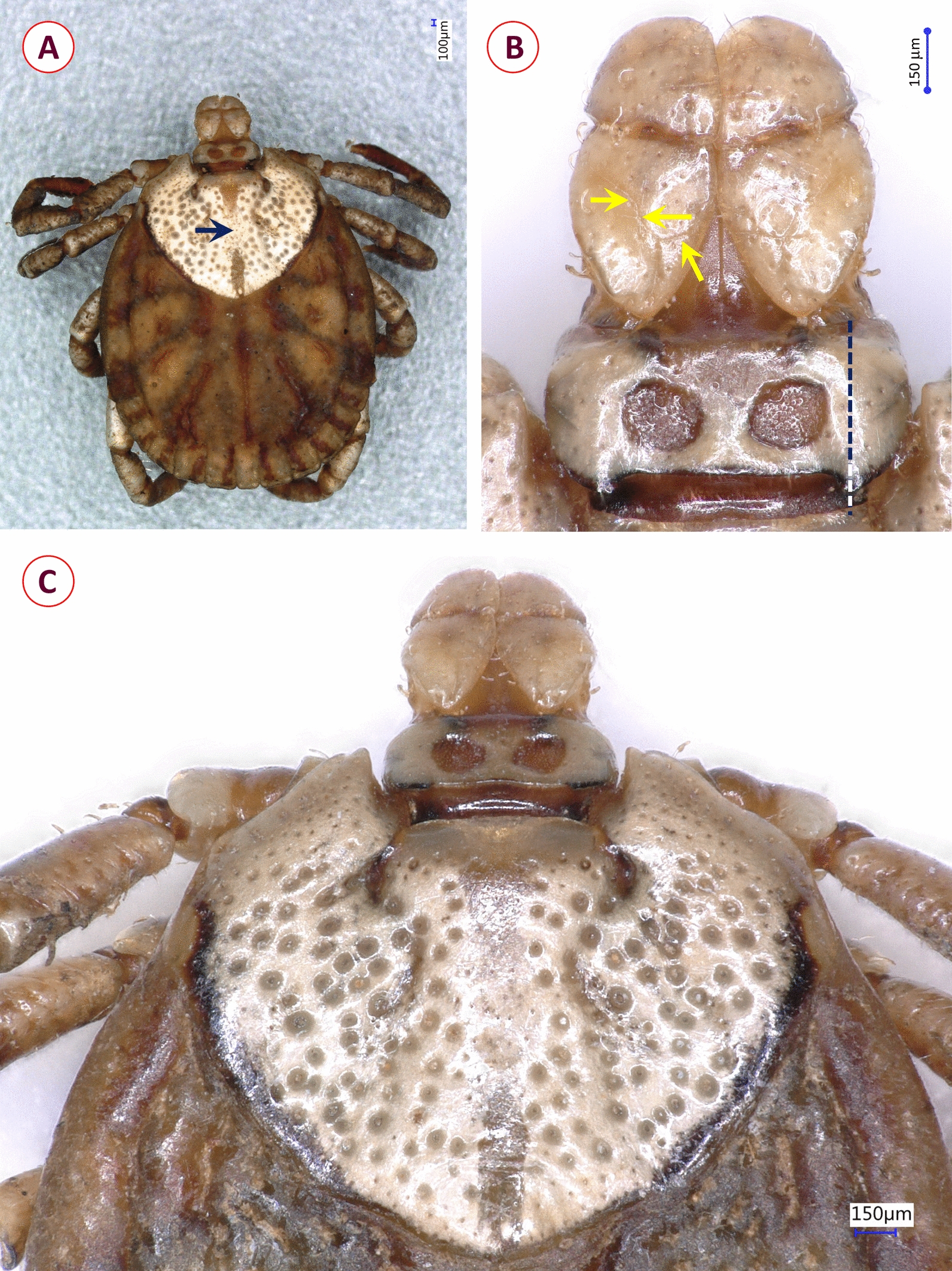
Dorsal view showing morphological characters of *Dermacentor pseudotamokensis* sp. nov. female holotype collected in Vietnam: **A** habitus (arrow: longitudinal, dark medial lane, lacking whitish enamel covering, discontinuous at mid-third); **B** perpendicular view of basis capituli, palps (arrows: delicate anteriolaterally directed groove is S-shaped in middle, joining with a short transverse groove at mid-length of palpal segment II; dashed line: longitudinal axis of cornua closer to porose area than to lateral edge of basis); **C** perpendicular view of scutum

**Fig. 8:**
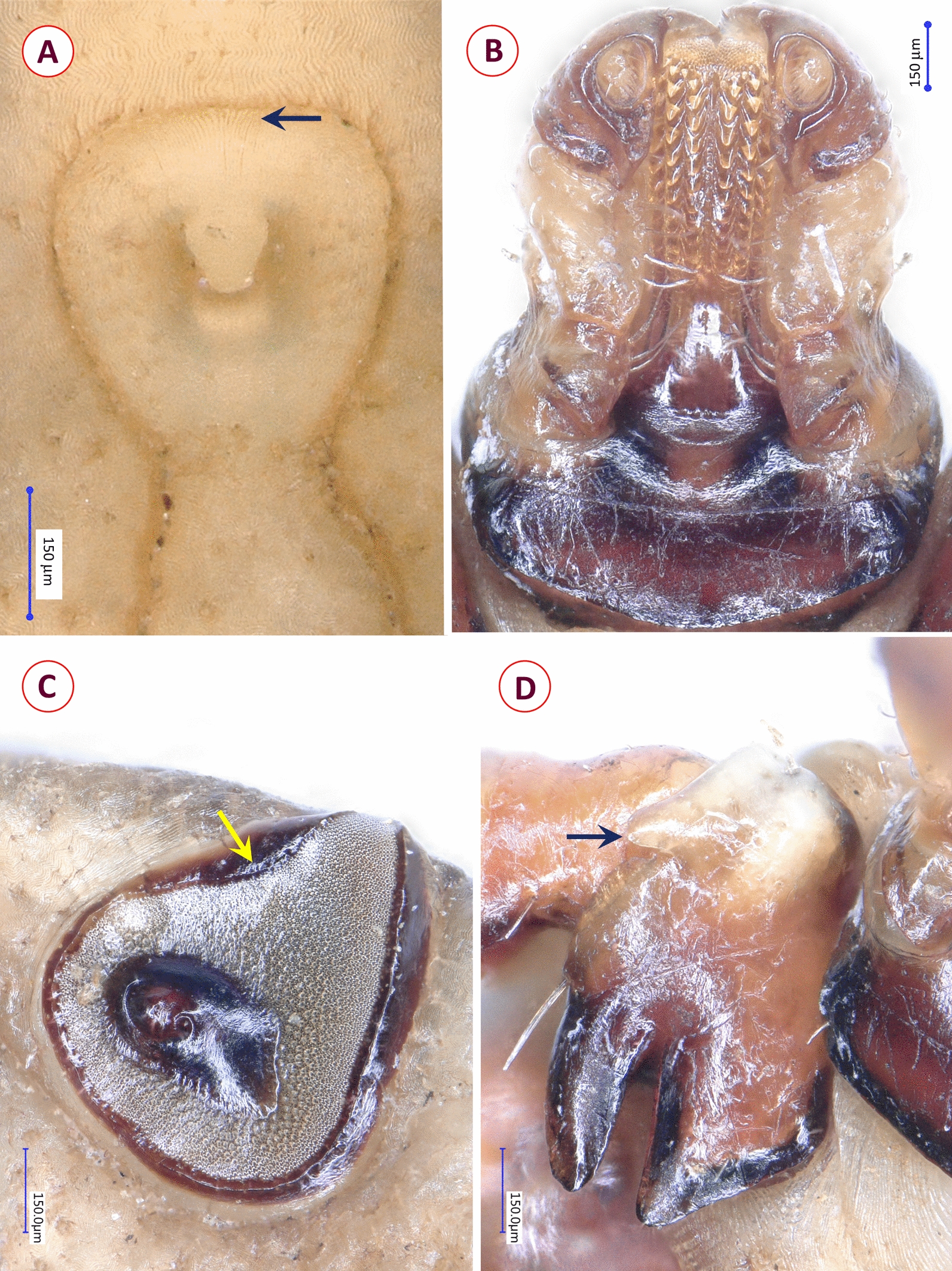
Ventral view showing morphological characters of *Dermacentor pseudotamokensis* sp. nov. female holotype collected in Vietnam: **A** genital aperture and anterior part of genital groove (arrow: surface between preatrial fold and anterior genital groove longitudinally striated); **B** basis capituli, hypostome and palps; **C** spiracular plate (arrow: anteriodorsal concavity of spiracular plate margin, flanking the area with pores, is deep with straight edges); **D** coxa I (arrow: anterior spur)

*Female*: [Based on the holotype; parameters provided in mm; Figs. 7, 8]. Length of idiosoma (from the half point between scapular apices to the middle of posterior margin) 4.7, width 3.8, ratio of idiosomal length/width 1.24 (Fig. 7A).

Scutum heart-shaped, broadest at anterior quarter, posteriorly rounded, with whitish enamel covering except lateral and posterior margins (Fig. 7C). Length of scutum 2.1, maximum width 2.65, ratio length/width 0.8. Eyes at maximum width (anterior third of length). On the scutum short, curved S-shaped cervical grooves, consisting of two parts: anteriorly as deep cervical pits, posteriorly shallow. Scutal punctuations scattered, small to large, deep; the former anteriolaterally, medially and posteriorly (Fig. 7C). Longitudinal, dark medial lane (lacking whitish enamel covering) discontinuous at mid-third (Fig. 7A). Scutal setae not visible.

Alloscutum with short (0.05) sparse hair covering, scattered punctuations. Idiosoma with sparse, minute (0.03–0.04) hair covering ventrally. Genital aperture reverse bell-shaped, 0.09 long, 0.1 wide, with slightly converging anterior margins, convexity in mid-length (with narrow semioval sclerites) and posteriorly rounded (Fig. 8A), situated between coxae II. Preatrial fold not bulging. Surface between preatrial fold and anterior genital groove longitudinally striated (Fig. 8A). Genital groove anteriorly slightly curved, convex, gradually diverging backwards until the level of anterior end of genital aperture; with concavity behind level of coxae IV. Spiracular plates asymmetrical, pear-shape, dorsal tapering process long, opening broad oval, surrounded by trapezoid area without aeropyles. Anteriodorsal concavity of spiracular plate margin (flanking the area with pores) deep, with straight edges (Fig. 8C). Caudally 11 festoons on idiosoma.

Length of gnathosoma (from palpal apices to posterior margin of basis capituli) 1.26, width of basis capituli dorsally 0.97. Ratio of gnathosomal length to basis capituli width 1.3. Length of basis capituli (from base of hypostome to posterior margin of basis capituli) 0.45, ratio of width to length of basis capituli 2.2. Shape of basis rectangular, its sides diverging and then converging backwards (Fig. 7B). Caudolateral edge of basis continuing in small, backward-pointing asymmetrical cornua. Longitudinal axis of cornua closer to porose area than to lateral edge of basis (Fig. 7B). Porose areas small, elliptical, breadth 0.2, interval narrow (0.12). Posterior edge of ventral basis broad V-shaped, showing transverse groove (Fig. 8B).

Palps (dorsal view) short, anteriorly rounded, posteriorly tapering to caudolateral spur-like extensions, with whitish enamel covering, length 0.8, maximum width 0.4, ratio length/width 2. Palpal hairs dorsally and medially/laterally few, short (0.06). Palpal segment II dorsally 0.5 long, anteriorly broadening, with a delicate anteriolaterally directed groove (S-shaped in middle) and a short transverse groove at mid-length (Fig. 7B). Palpal segment III 0.32 long, with caudal concavity (Fig. 7B). Palps ventrally showing caudally directed triangular spur on segments I and III; four plus four long medial hairs on segments I (0.13), II (0.11). Hypostome reaching anterior edge of palpal segment IV, anteriorly broadening, with blunt tip and dental formula 3/3 (Fig. 8B).

Legs with whitish enamel covering. Coxae I trapezoid, with deep incision separating two closely situated, nearly parallel spurs, with slightly concave medial edges. Coxa I anteriorly to internal spur trapezoid, external spur claw-shaped, laterally with a relatively long (0.2) hair. Anterior spur on coxa I caudolaterally directed (Fig. 8D). Two spurs on each coxae: internal spur shorter than external spur on coxa II; internal and external spurs equal on coxae III–IV. Trochanter I with caudal spur.

### Differential diagnosis

The internal and external spurs on coxa I are widely separated in adults of *Dermacentor filippovae*, *D. limbooliati*, *D. pasteuri*, *D. auratus*, *D. compactus* and *D, pseudocompactus* whereas closely situated in the female of *D. pseudotamokensis* sp. nov. Large punctuations are scarce in the posterior third of the scutum in the females of *Dermacentor bellulus* and *D. taiwanensis*, while these are numerous in *D. pseudotamokensis* sp. nov. Females of several Southeast Asian *Dermacentor* species have broad or rounded U-shaped genital apertures, as exemplified by *Dermacentor tricuspis* and *D. bellulus*, whereas for *D. steini* (Supplementary Fig. 1), *D. taiwanensis* or *D. falsosteini*, and *D. laothaiensis*, the shape is acute-angled (resembling broad or narrow V) without posterior rounding as in *D. pseudotamokensis* sp. nov. Furthermore, in most of these *Dermacentor* spp. the anterior, transverse part of the genital groove is straight (Supplementary Fig. 1), except *D. pseudocompactus* (for which the female is unknown), *D. filippovae* (where it is convex) and *D. bellulus* (where it is concave), unlike in *D. pseudotamokensis* sp. nov. where it is slightly curved, convex.

The species most closely resembling *D. pseudotamokensis* sp. nov. is *D. tamokensis* (Supplementary Fig. 2), the female of which has a similar shape of the genital aperture, but the area anterior to the preatrial fold is smooth behind the genital groove; the basis capituli is longer (width to length ratio is 1.8–2.1, vs 2.2 in *D. pseudotamokensis* sp. nov.) (Supplementary Fig. 2). The dorsal transverse groove of palpal article II is situated at the level of posterior third in *D. tamokensis*, whereas at half-length in *D. pseudotamokensis* sp. nov. Cornua is symmetrical, its longitudinal axis closer to the margin of basis than to porose area in *D. tamokensis*, unlike in *D. pseudotamokensis* sp. nov. (Supplementary Fig. 2). On the scutum of female *D. tamokensis*, the central, longitudinal brown lane (without whitish enamel covering) usually extends from the anterior to the posterior scutal margin, unlike in *D. pseudotamokensis* sp. nov. where it is interrupted in the middle (Supplementary Fig. 2). Fine punctuations are more numerous in the caudal and the central areas of scutum in *D. pseudotamokensis* sp. nov. than in *D. tamokensis*. In the spiracular plates, the anteriodorsal edge of area with pores is deeply concave, straight in *D. pseudotamokensis* sp. nov. but shallower and curved in *D. tamokensis* (Supplementary Fig. 2).

## Discussion

This study aimed to contribute to the barcoding of tick species indigenous to Vietnam, which was recently initiated [2]. Specimens of all three tick species examined here are not only provided with sequences of two molecular genetic markers but also illustrated with high-quality digital pictures for the first time in the whole region of Southeast Asia.

This study reports the first mitochondrial gene sequences of the Asian rodent tick, *I. granulatus*, from any of the countries of the Indochinese Peninsula. This tick species can be found in various ecoregions of the Australasian, Oriental and eastern part of the Palearctic Zoogeographic Regions [3]. Results of molecular analyses in this study indicated that, unexpectedly, the most different haplotypes of *I. granulatus* were found within 500 (in Southern China) and in 1000 km (in Malaysia) from the collection site of this species in Vietnam, whereas its near identical haplotypes were reported as far as 2000 km from Vietnam, in Pakistan and in Japan. The most likely explanation for this phenomenon is the host preference of this tick species. *Ixodes granulatus* does not only associate with hosts that are restricted in their geographical range (e.g. wild rodent species: [20]). It also frequently infests humans [21] as well as animal hosts that are widespread owing to historical, human-driven dispersal events, i.e. black rats [22], and even more often brown rats [23], which were dispersed from Southeast Asia towards the Western Palearctic [24]. It is also found on birds, allowing long-distance geographical dispersal [25]. In support of the need of mapping its geographical relationships, numerous zoonotic pathogens have been detected in *I. granulatus*, suggesting its significant role in transmitting vector-borne pathogens to humans [26].

*Haemaphysalis bispinosa* has Oriental distribution [3]. This tick species is new to the fauna of Vietnam, increasing the number of indigenous species to 65. As revealed here, mitochondrial haplotypes of *H. bispinosa* are shared in the whole of South and Southeast Asia, probably owing to human activity, i.e. transportation of its main hosts, such as domestic ruminants and dogs [3]. Another important factor in this respect could be dispersal events by birds, which are also among the main hosts of this tick species [3]. Accordingly, half a century ago, *H. bispinosa* was considered to be extending its geographical range eastward; it was reported to be imported [27] and recently well-established in Malaysia and Indonesia [28]. While the microbiome of *H. bispinosa* is known to include several pathogenic, even zoonotic microorganisms, some in high ratios [29], the extent of its Southeast Asian distribution is debated, because it is easily confused with *Haemaphysalis longicornis* [3]. In particular, *H. bispinosa* was reported in China, including southern parts of the country close to northern Vietnam (sampled here), but these records were later deemed incorrect [30]. In light of the present results, however, it seems to be possible, and should be re-evaluated if this tick species occurs in southern China.

The genus *Dermacentor* has been found in all zoogeographical regions but not on remote islands, and no endemic species have been recorded from Australasia [1]. The highest number of species, i.e. 16 from this single genus (representing 36% of all *Dermacentor* species), has been recorded from the Oriental region [1]. Probably contributing to their geographical isolation, *Dermacentor* sp. ticks, as in other zoogeographical regions, are rarely transported by birds in Oriental Asia (e.g. [25]). From this genus, only *D. taiwanensis* was reported from birds in the region of Vietnam [2], in particular in Taiwan [31]. However, for most *Dermacentor* spp. indigenous in Vietnam, wild mammals (usually Suidae) are typical hosts, and they can infest humans [2], indicating their medical importance. With *D. pseudotamokensis* sp. nov., the number of ixodid species in the fauna of Vietnam is 66.

Morphological key characters and phylogenetic relationships of *D. tamokensis* from Vietnam raise the possibility that there might be some confusion in identifying ticks from this genus in the region. Notably, ticks reported from China under the name *D. steini* clustered with *D. pseudotamokensis* sp. nov., suggesting that they belong to the same species. The possible misidentification of *D. steini* in the above context cannot be verified as neither the identification key nor any tick illustrations are provided in the original paper [32] to describe how the ticks were identified. Thus, and based on its partial sequences (*cox*1, 16S) obtained here, the complete mitochondrial genome of *D. pseudotamokensis* sp. nov. is probably also available in GenBank under the name *D. steini* (OM368300, OP383032) [33]. To prevent misidentifications, this study is also intended as a plea for barcoding of all *Dermacentor* species indigenous to Southeast Asia, especially if we consider the missing sequence data in GenBank for recently described new species in or near Vietnam (e.g. *Dermacentor limbooliati*, *D. filippovae*).

## Conclusions

Based on two mitochondrial markers, *H. bispinosa* shows genetic homogeneity in Southern-Southeastern Asia. This can be explained by its frequent association with domestic ruminants and dogs, which are frequently transported hosts. However, *I. granulatus* has a mixed, mosaic-like geographical pattern of haplotypes, probably because it frequently associates with both synanthropic and wild rodents. This tick species is also recorded here parasitizing lorises for the first time in Vietnam and its region. Based on the morphological and phylogenetic analyses, *D. pseudotamokensis* sp. nov. is recognized and described here as a new tick species that was almost certainly misidentified hitherto as *D. steini*. This draws the attention of taxonomists to the need of barcoding all *Dermacentor* spp. in Southeast Asia.

## Supplementary Information

Supplementary material 1: Figure 1. Differences in the genital aperture and preatrial genital groove of *Dermacentor pseudotamokensis* sp. nov. and *Dermacentor steini*. Arrows indicate distinguishing characters described in the text.

Supplementary material 2: Figure 2. Differences in the scutum, palps, basis capituli, coxa I, genital aperture and spiracle opening of *Dermacentor pseudotamokensis* sp. nov. and *Dermacentor tamokensis*. Arrows indicate distinguishing characters described in the text. Dashed line marks the longitudinal axis of cornua.

Supplementary material 3: Table 1. Geographical comparison of *Ixodes granulatus* sequences from Vietnam with those of conspecific ticks in GenBank.

## Data Availability

The sequences obtained during this study are deposited in GenBank under the following accession numbers. Cox1 gene: PQ439197-PQ439201, 16S rRNA gene: PQ453065-PQ453069. All other relevant data are included in the manuscript and the supplementary material or are available upon request by the corresponding author.
