# Supplementary material for: First report of Haemaphysalis bispinosa, molecular-geographic relationships of Ixodes granulatus and a new Dermacentor species from Vietnam
Source: Parasit Vectors. 2025 Jan 23;18:21. doi: 10.1186/s13071-024-06641-7 (PMC11755799; doi:10.1186/s13071-024-06641-7)
Supplement: Supplementary file 1 — Supplementary material 1: Figure 1. Differences in the genital aperture and preatrial genital groove of Dermacentor pseudotamokensis sp. nov. and Dermacentor steini. Arrows indicate distinguishing characters described in the text. [file 13071_2024_6641_MOESM1_ESM.pdf]

Supplementary Figure 1

genital aperture and preatrial genital groove

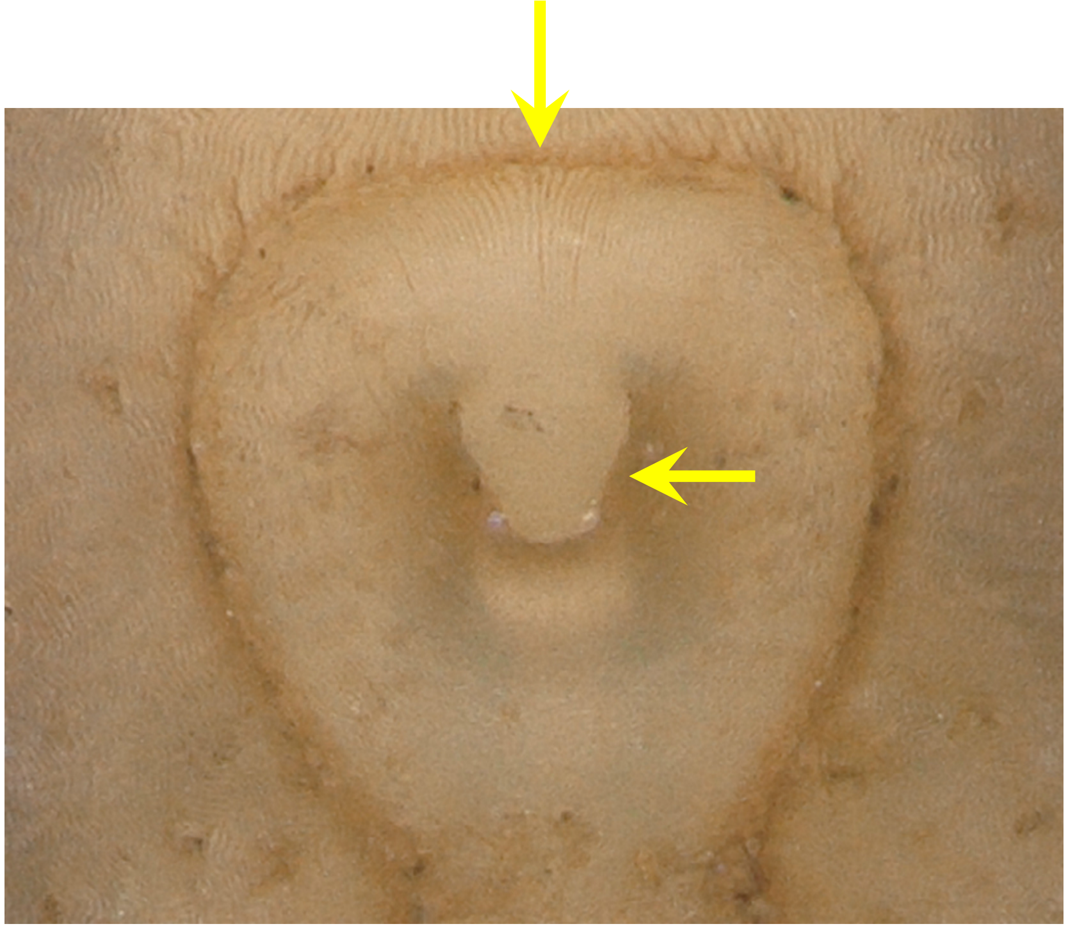

Dermacentor pseudotamokensis
(this study)

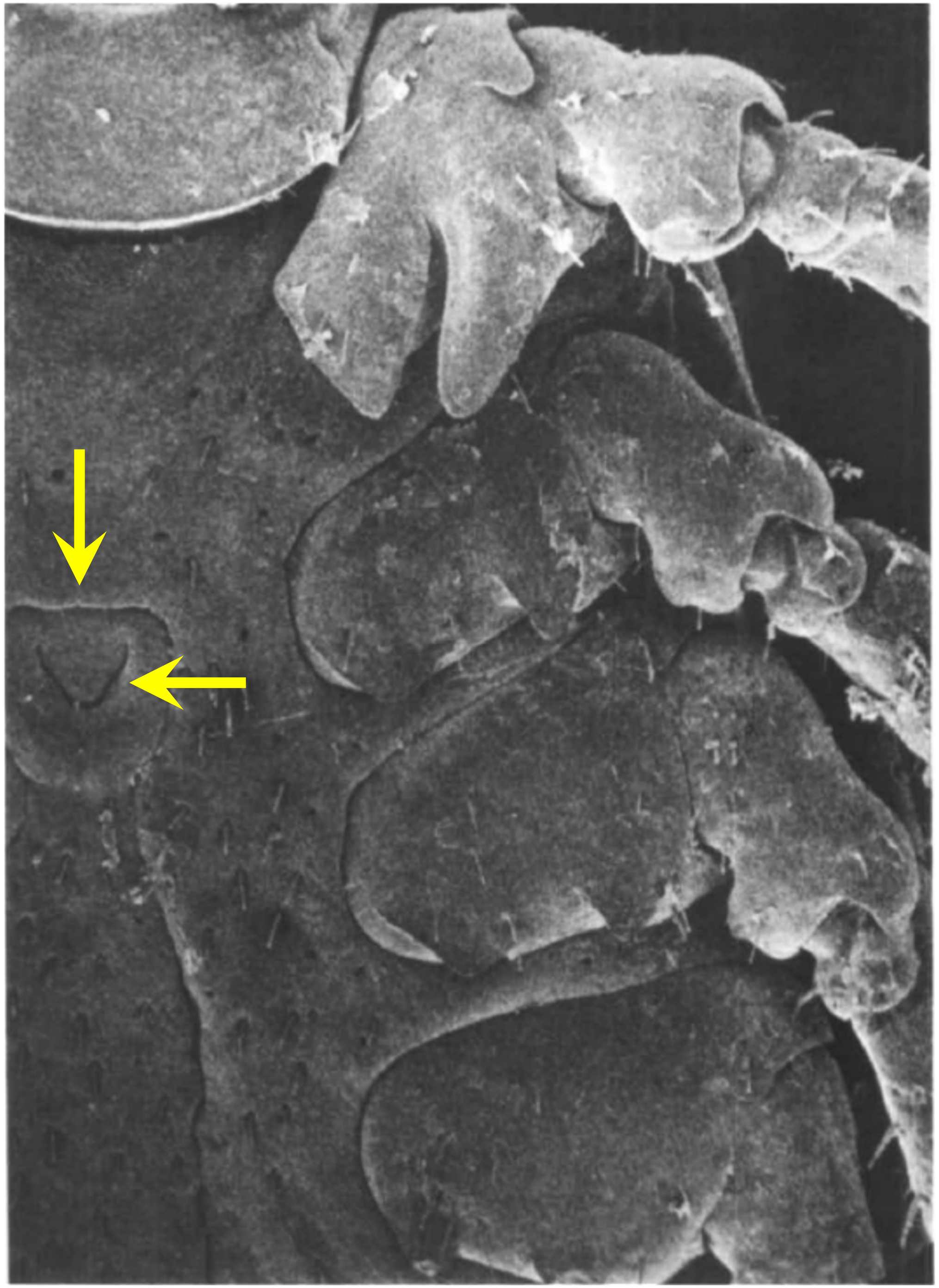

Dermacentor steini

(Wassef HY, Hoogstraal H. *Dermacentor* (*Indocentor*) *steini* (Acari: Ixodoidea: Ixodidae): identity of male and female. J Med Entomol. 1986;23:532-7. doi: 10.1093/jmedent/23.5.532.)
