# Supplementary material for: First report of Haemaphysalis bispinosa, molecular-geographic relationships of Ixodes granulatus and a new Dermacentor species from Vietnam
Source: Parasit Vectors. 2025 Jan 23;18:21. doi: 10.1186/s13071-024-06641-7 (PMC11755799; doi:10.1186/s13071-024-06641-7)
Supplement: Supplementary file 2 — Supplementary material 2: Figure 2. Differences in the scutum, palps, basis capituli, coxa I, genital aperture and spiracle opening of Dermacentor pseudotamokensis sp. nov. and Dermacentor tamokensis. Arrows indicate distinguishing characters described in the text. Dashed line marks the longitudinal axis of cornua. [file 13071_2024_6641_MOESM2_ESM.pdf]

Supplementary Figure 2

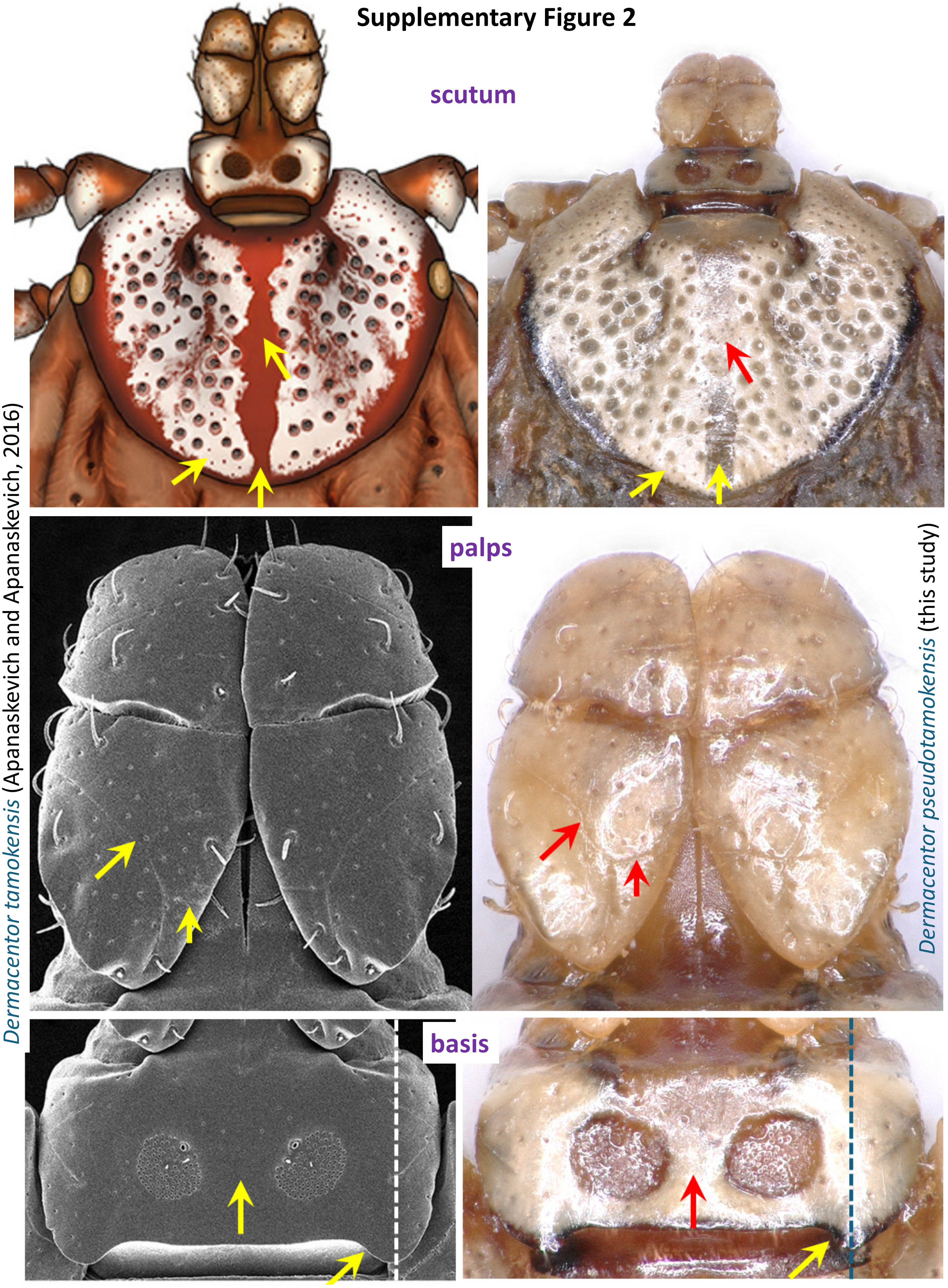

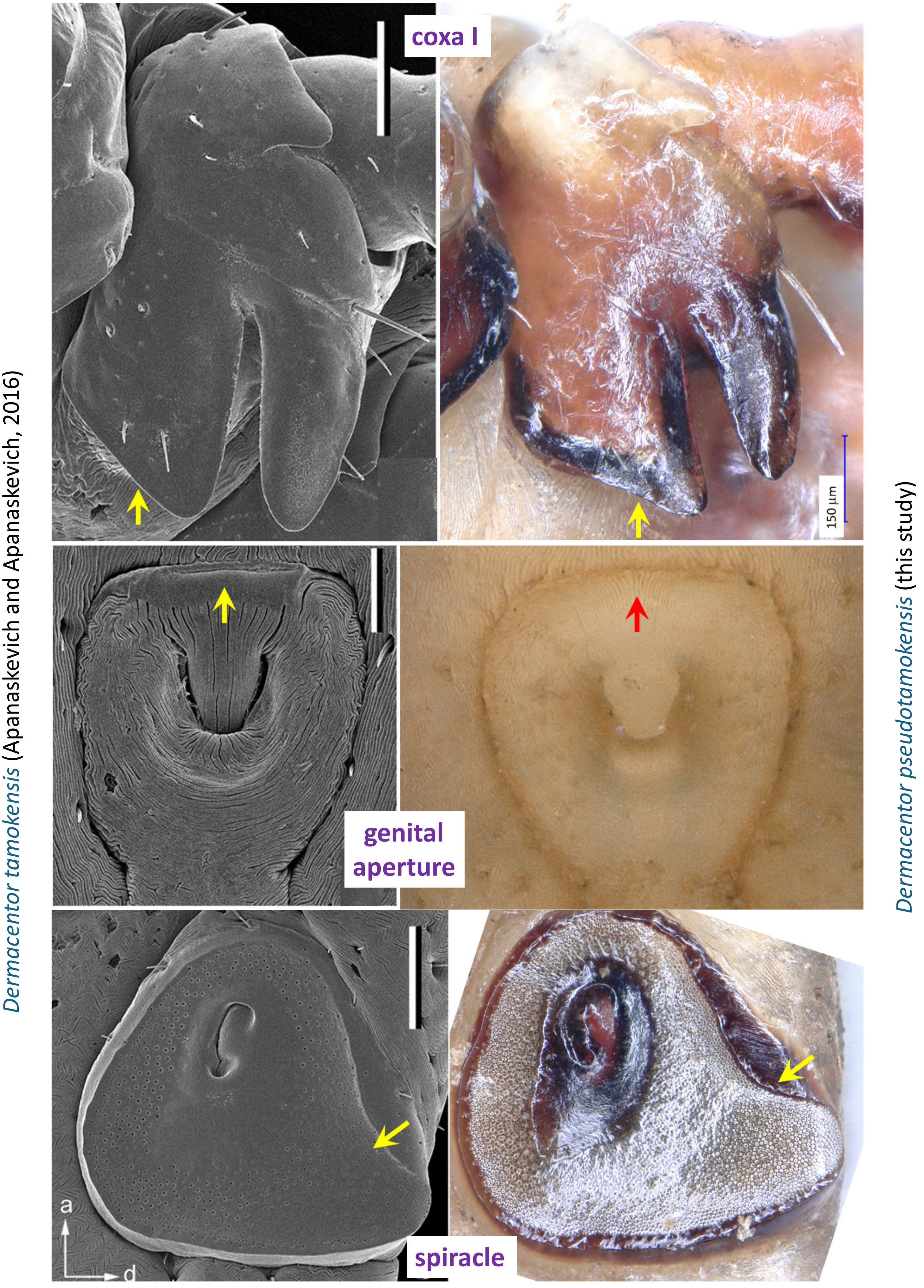

Dermacentor tamokensis (Apanaskevich and Apanaskevich, 2016)
coxa I
150 µm
genital aperture
spiracle
a
d
Dermacentor pseudotamokensis (this study)
