# Supplementary material for: First report of Haemaphysalis bispinosa, molecular-geographic relationships of Ixodes granulatus and a new Dermacentor species from Vietnam
Source: Parasit Vectors. 2025 Jan 23;18:21. doi: 10.1186/s13071-024-06641-7 (PMC11755799; doi:10.1186/s13071-024-06641-7)
Supplement: Supplementary file 3 — Supplementary material 3: Table 1. Geographical comparison of Ixodes granulatus sequences from Vietnam with those of conspecific ticks in GenBank. [file 13071_2024_6641_MOESM3_ESM.pdf]

Supplementary Table 1. Geographical comparison of cox1 and 16S rRNA sequences from *Ixodes granulatus* collected in Vietnam (VQ15 and VQ14, respectively) with conspecific ticks in GenBank.

Country	Province/Island	County/City	COI Acc.no	bp/bp (%)	16S Acc.no	bp/bp (%)
China	Hajnan	Chengmai	OM368272	642/642 (100%)	OM368272	425/426 (99.8%)
China	Jiangxi		MG721046	641/642 (99.8%)		
India	Mizoram		OQ699275	641/642 (99.8%)		
China*	GuangDong	ZhongShan	KM497429	640/642 (99.7%)		
China	Hubei	Daye	OM368258	639/642 (99.5%)	OM368258	423/426 (99.3%)
China*	GuangDong	ZhongShan	KM497437	639/642 (99.5%)		
Japan			AB231673	639/642 (99.5%)		
China*	GuangDong	ZhongShan	KM497433	639/642 (99.5%)		
China	Jiangxi		MG721045	638/642 (99.4%)		
China*	GuangDong	ZhongShan	KM497432	637/642 (99.2%)		
China	Yunnan		NC_061226	606/642 (94.4%)	NC_061226	419/429 (97.7%)
China	Jiangxi		MG721051	510/525 (97.1%)		
China	Zhejiang		JF758633	452/453 (99.8%)		
China	Zhejiang		JQ625686	452/454 (99.6%)		
China	Zhejiang	Wenzhou	JQ625690	452/454 (99.6%)		
Taiwan	Lanyu				EF427898	426/426 (100%)
China	Jiangxi				MG696716	422/422 (100%)
China	Jiangxi				MG696715	422/422 (100%)
China	Jiangxi				MG696714	421/422 (99.8%)
China	Jiangxi				MG696718	421/422 (99.8%)
Taiwan					DQ093302	424/428 (99.1%)
Taiwan					DQ093303	424/428 (99.1%)
Taiwan					DQ093307	424/428 (99.1%)
Taiwan					DQ093305	422/426 (99.1%)
Taiwan					DQ093291	423/427 (99.1%)
Taiwan					DQ093304	418/420 (99.5%)
Taiwan					DQ093306	423/427 (99.1%)
Taiwan					DQ093309	422/426 (99.1%)

Taiwan					DQ093308	424/429 (98.8%)
Taiwan					DQ093301	422/427 (98.8%)
Taiwan					DQ093290	415/417 (99.5%)
Taiwan					DQ002994	426/433 (98.4%)
Taiwan					DQ002996	426/433 (98.4%)
Taiwan					DQ093300	414/417 (99.3%)
Taiwan					DQ093289	409/411 (99.5%)
Taiwan		Nantou			OK047514	403/403 (100%)
Taiwan					DQ093294	407/409 (99.5%)
Taiwan					DQ093295	407/409 (99.5%)
Japan	Okinawa				AB819235	403/403 (100%)
Malaysia					MT914180	419/427 (98.1%)
Japan		Tokyo			AB819237	402/403 (99.8%)
Japan	Okinawa				AB819236	402/403 (99.8%)
Taiwan					DQ093287	410/415 (98.8%)
Taiwan					DQ093293	403/407 (99%)
Taiwan					DQ093286	402/407 (98.8%)
Taiwan					DQ093292	401/406 (98.8%)
Taiwan					DQ093296	400/405 (98.8%)
Taiwan					DQ002995	418/430 (97.2%)
Malaysia					MT914179	415/428 (97%)
Pakistan					OR905655	389/390 (99.7%)
Pakistan					OR905655	402/411 (97.8%)
Pakistan					OR905656	380/380 (100%)
Malaysia					LC602428	396/405 (97.8%)
Malaysia					LC602431	396/405 (97.8%)
Malaysia					LC602424	395/404 (97.8%)
Malaysia					LC602432	396/405 (97.8%)
Malaysia					LC602427	396/405 (97.8%)
Malaysia					LC602422	395/404 (97.8%)
Malaysia					LC602429	396/405 (97.8%)

Malaysia					LC602426	395/404 (97.8%)
Malaysia					LC602430	396/405 (97.8%)
Malaysia					LC602423	395/404 (97.8%)
Malaysia					LC602425	394/406 (97%)
China	Jiangxi				MG696717	340/340 (100%)

*medical record, probably originating in the designated area
